# SIRT2 in metabolism, aging, and longevity

**DOI:** 10.3389/fmolb.2026.1990670

**Published:** 2026-10-12

**Authors:** Menghui Li, Yihuan Dong, Wen Ren, Weixia Dong, Shaoping Ji

**Affiliations:** Henan Provincial Research Center of Engineering Technology for Nuclear Protein Medical Detection, Zhengzhou Health College, Zhengzhou, Henan Province, China

**Keywords:** aging, energy metabolism, lifespan, metabolic homeostasis, SIRT2

## Abstract

SIRT2, an NAD^+^-dependent deacetylase, is increasingly recognized as an important regulator of cellular metabolism and aging. By deacetylating diverse substrates, SIRT2 influences glucose metabolism, lipid metabolism, mitochondrial function, cytoskeletal dynamics, oxidative stress, and inflammatory responses, thereby contributing to cellular metabolic homeostasis and aging. Emerging evidence suggests that age-related alterations in NAD^+^ metabolism may affect SIRT2 activity and disrupt these processes, potentially promoting metabolic dysfunction, mitochondrial impairment, cellular senescence, and chronic inflammation. In addition, the effects of SIRT2 on aging appear to be highly context- and tissue-dependent, and its precise contribution to organismal longevity remains incompletely understood. In this review, we primarily focus on the role of SIRT2 in aging and longevity through the regulation of energy metabolism, with particular emphasis on the underlying molecular mechanisms and its interactions with metabolic and stress-responsive pathways. Understanding these mechanisms and regulations may provide new insights into SIRT2 biology and potential strategies for maintaining metabolic homeostasis and promoting healthy aging, even applications in clinic practice.

## 1 Introduction

Population aging is rapidly emerging as one of the greatest challenges to global public health (Gianfredi et al., 2025). Ageing is a complex biological process, and its onset and progression involve a variety of mechanisms, including imbalances in energy metabolism, the accumulation of oxidative stress, chronic inflammation, and reduced genomic stability (López-Otín et al., 2013). In recent years, there has been a growing body of research indicating that the state of energy metabolism not only determines cellular function but also is increasingly recognized as a fundamental determinant in regulating the ageing process and lifespan in living organisms (Bartke et al., 2021). Sirtuins are a family of NAD^+^-dependent deacetylases and ADP-ribosyltransferases that function as central regulators of energy metabolism, cellular stress responses, and aging (Houtkooper et al., 2012; Winnik et al., 2015).

Sirtuin family is a group of seven protein enzymes (SIRT1–SIRT7) found in mammals. Among these, SIRT2 is primarily localized in the cytoplasm with shuttle to nuclear and plays a crucial role in the regulation of glycolysis, fatty acid metabolism, and mitochondrial function (Wang et al., 2019). As an NAD^+^-responsive metabolic sensor, SIRT2 responds to changes in intracellular NAD^+^ levels, thereby participating in the regulation of oxidative stress, inflammatory responses, and cell fate determination. Emerging evidence suggests that SIRT2 integrates metabolic adaptation with cellular stress responses, thereby linking energy metabolism to healthy aging and lifespan regulation (Szczęsny-Małysiak et al., 2026).

However, the specific mechanisms by which SIRT2 contributes to the regulation of organismal lifespan through the control of energy metabolism remain incompletely understood. Although SIRT1 has been extensively investigated as a longevity-associated metabolic regulator, SIRT2 predominantly localizes to the cytoplasm and regulates a distinct repertoire of metabolic enzymes and cytoskeletal proteins, suggesting that it may influence aging through mechanisms different from those of other sirtuins. Nevertheless, considerably less attention has been paid to the role of SIRT2 in coordinating metabolic pathways that determine organismal lifespan.

In this review, we summarize current evidence regarding the role of SIRT2 in metabolic regulation and discuss how SIRT2-mediated metabolic pathways contribute to aging and lifespan regulation. We also highlight the therapeutic potential of targeting SIRT2 for promoting healthy aging and preventing age-related diseases.

## 2 SIRT2 as a metabolic sensor

### 2.1 NAD^+^-dependent deacetylase and its relationship with longevity-associated sirtuins

The sirtuin family was originally identified through studies of the yeast Silent Information Regulator 2 (Sir2), which was first characterized as an NAD^+^-dependent histone deacetylase linking cellular metabolism to chromatin silencing and lifespan regulation (Imai et al., 2000). This seminal discovery laid the foundation for the identification of mammalian sirtuins (SIRT1–SIRT7). Among them, SIRT2 belongs to the class III histone deacetylase (HDAC) family, and its conserved catalytic core domain of approximately 275 amino acids contains a Rossmann-fold domain responsible for NAD^+^ binding and a Zn^2+^-binding domain that maintains protein structural stability (Frye, 2000). Its N- and C-terminal extensions regulate subcellular localization and catalytic activity (Moniot et al., 2013). Although SIRT2 is predominantly localized in the cytoplasm, it accumulates in the nucleus during the G2/M phase of the cell cycle (Vaquero et al., 2006; Dryden et al., 2003).

There is growing evidence that biological ageing is closely associated with a decline in SIRT1 expression and activity, and that this change may contribute to the onset and progression of many age-related diseases. Conversely, activation of SIRT1 has been shown improves age-related cellular abnormalities, such as mitochondrial dysfunction, oxidative stress and cellular senescence (Abdulkhaliq et al., 2025). Further investigation of the molecular mechanisms underlying SIRT2-mediated metabolic regulation may therefore provide important insights into its potential contribution to healthy aging and longevity.

### 2.2 SIRT2 maintains cellular energy homeostasis

Energy metabolism provides the essential energy required for diverse cellular activities and maintains cellular homeostasis, thereby playing a fundamental role in supporting normal physiological functions. Disruption of energy metabolic homeostasis is closely associated with the development and progression of various diseases, including neurodegenerative disorders, autoimmune diseases, metabolic disorders, and cancer (Liu et al., 2025). Energy homeostasis refers to the ability of cells to maintain a dynamic equilibrium between energy supply, energy production and energy consumption under conditions where nutrient supply and metabolic demands are constantly changing. The maintenance of this equilibrium depends on the dynamic sensing of intracellular metabolic states and the coordinated control of downstream adaptive responses (Cantó et al., 2015). The sirtuin family is considered to be important metabolic sensors capable of linking changes in intracellular metabolic status with adaptive metabolic control. Among the members of the mammalian sirtuin family, SIRT2 occupies a unique position in the regulation of cellular metabolism, as it is primarily localized in the cytoplasm and has a broad spectrum of non-histone substrates. SIRT2’s ability to sense metabolic changes stem primarily from its NAD^+^ dependence.

As a key metabolic product involved in cellular redox reactions and energy metabolism, intracellular NAD^+^ levels fluctuate dynamically in response to changes in nutritional status, mitochondrial function and cellular energy demand (Figure 1). Consequently, SIRT2’s NAD^+^-dependent deacetylase activity enables it to respond dynamically to changes in the cell’s metabolic state (Yi et al., 2026). By regulating the acetylation status of target proteins, SIRT2 influences their function, stability and intracellular localization, thereby enabling cells to dynamically adjust their metabolic activity and maintain energy homeostasis. Collectively, these features suggest that SIRT2 may function as a metabolic checkpoint that links changes in cellular energy status to adaptive metabolic responses, thereby contributing to the maintenance of energy homeostasis and cellular function during metabolic stress and aging.

**FIGURE 1:**
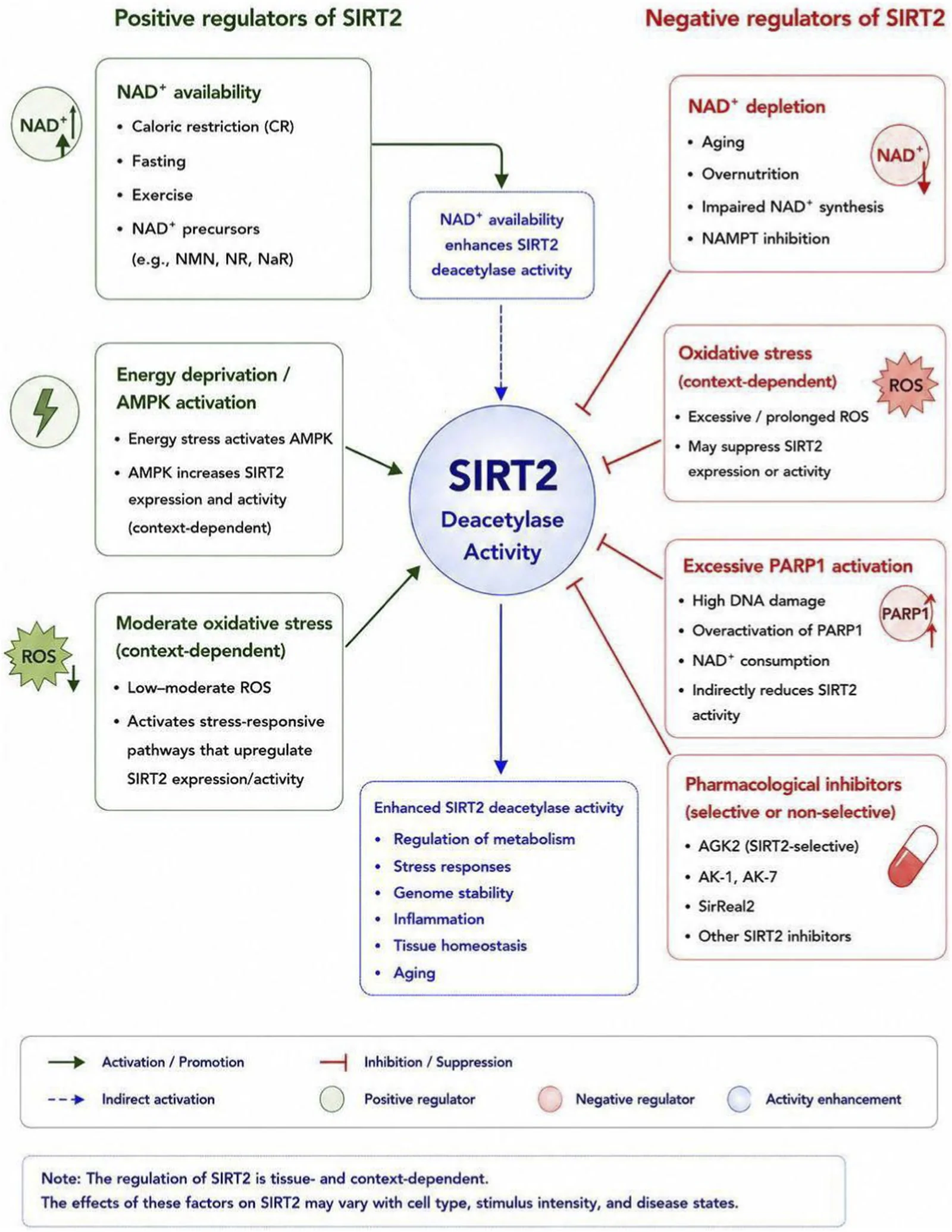
Positive and negative regulators of SIRT2. SIRT2 activity is regulated by cellular metabolic status, NAD^+^ availability, energy stress, and oxidative stress. Increased NAD^+^ availability, caloric restriction, fasting, exercise, and moderate oxidative stress may enhance SIRT2 activity, whereas NAD^+^ depletion, excessive oxidative stress, excessive PARP1 activation, and pharmacological inhibitors may suppress SIRT2 function. The regulation of SIRT2 is highly tissue- and context-dependent, and the effects of individual regulators may vary according to cell type, metabolic state, stimulus intensity, and disease conditions.

## 3 Metabolic pathways regulated by SIRT2

### 3.1 Regulation of glucose metabolism

Glucose metabolism provides the primary source of ATP for most mammalian cells and is therefore indispensable for maintaining cellular energy homeostasis and activity (DeBerardinis and Thompson, 2012). Accumulating evidence suggests that SIRT2 fine-tunes glycolytic flux through the site-specific deacetylation of key glycolytic enzymes, thereby influencing cellular energy homeostasis through context-dependent regulation of glycolytic metabolism.

SIRT2 promotes hepatic glucose uptake by deacetylating glucokinase regulatory protein (GKRP) at K126, thereby facilitating the glucose-dependent dissociation of GKRP from glucokinase. Hepatic SIRT2 overexpression improves hepatic glucose uptake and glucose tolerance in obese diabetic mice, whereas SIRT2 downregulation reduces hepatic glucose uptake and impairs glucopse tolerance (Watanabe et al., 2018). Among the glycolytic enzymes regulated by SIRT2, pyruvate kinase M2 (PKM2) has been the most extensively characterized. Notably, SIRT2 regulates PKM2 through site-specific deacetylation. SIRT2 enhances enzyme activity by deacetylating lysine 305 (K305) of PKM2 and promoting the formation of its active tetramer. Deficiency of SIRT2 increases the acetylation of PKM2 and inhibits its tetramerization, thereby reducing enzymatic activity. Consequently, the glycolytic metabolism of cancer cells is reprogrammed, promoting tumour proliferation (Park et al., 2016). In contrast, SIRT2-mediated metabolic reprogramming and inflammation play a crucial role in the development of neuropathic pain. Ononin, a compound isolated from various plants, increases SIRT2 expression in LPS-induced BV2 microglial cells and suppresses inflammation and glycolysis. This effect depends on SIRT2-mediated deacetylation at the K207 site of PKM2, which reduces PKM2 stability. Inhibition of SIRT2 attenuates the effects of ononin, promotes PKM2 acetylation, and consequently enhances the activation of the glycolytic pathway (Chen Q. et al., 2025). Together, these findings indicate that SIRT2 exerts distinct regulatory effects on PKM2 through deacetylation at different lysine residues.

The regulatory role of SIRT2 extends beyond PKM2. SIRT2 also targets phosphoglycerate mutase 2 (PGAM2), another key glycolytic enzyme, by deacetylating its K100 residue, thereby reducing protein stability and promoting its ubiquitination and degradation, which ultimately enhances aerobic glycolysis in hepatocellular carcinoma cells (Wa et al., 2025). Together, these findings indicate that SIRT2 regulates multiple nodes within the glycolytic pathway rather than acting on a single metabolic enzyme.

SIRT2 deacetylates and stabilizes the gluconeogenic enzyme phosphoenolpyruvate carboxykinase 1 (PEPCK1), thereby contributing to cellular responses to changes in glucose availability and regulating gluconeogenesis. Mechanistically, SIRT2-mediated deacetylation counteracts P300-dependent acetylation of PEPCK1, thereby reducing UBR5-mediated ubiquitination and degradation of PEPCK1 (Jiang et al., 2011).

Interestingly, the functional consequences of SIRT2-mediated PGAM2 K100 deacetylation appear to be context-dependent. Under oxidative stress, SIRT2-mediated deacetylation activates PGAM2 and contributes to NADPH homeostasis, whereas in hepatocellular carcinoma, the same deacetylation promotes PGAM2 ubiquitination and degradation, thereby enhancing aerobic glycolysis. Taken together, these findings advance our understanding of how SIRT2 coordinates glucose utilization and energy metabolism through site-specific deacetylation of multiple metabolic enzymes, with distinct deacetylation sites and cellular contexts contributing to substrate-specific metabolic regulation. Given the fundamental role of glucose metabolism in the ageing process, SIRT2-mediated regulation of glucose metabolism may represent one metabolic mechanism linking metabolic homeostasis to aging and lifespan regulation. Further studies are needed to identify additional substrates and elucidate the underlying mechanisms.

### 3.2 Regulation of lipid metabolism

Lipid metabolism plays a critical role in maintaining energy homeostasis and physiological function. Dysregulation of lipid metabolism contributes to metabolic disorders and age-related diseases, highlighting the importance of understanding its molecular regulation during aging (Corvera et al., 2026). SIRT2 is an important regulator of lipid metabolism, exerting its regulatory functions across multiple metabolic tissues through the site-specific deacetylation of key metabolic enzymes and transcription factors. SIRT2 deacetylates ATP-citrate lyase (ACLY), thereby reducing its stability and promoting its ubiquitination and degradation, ultimately limiting *de novo* lipid synthesis (Lin et al., 2013). SIRT2 binds to and deacetylates hepatocyte nuclear factor 4α (HNF4α) at Lys458, thereby enhancing HNF4α protein stability. Through an HNF4α-dependent mechanism, SIRT2 alleviates insulin resistance, hepatic steatosis, and inflammatory responses, whereas SIRT2 deficiency exacerbates these metabolic abnormalities (Ren et al., 2021). In the heart, abnormalities in lipid metabolism are a key factor in the development of diabetic cardiomyopathy (DCM). Mechanistically, SIRT2 deacetylates carnitine palmitoyltransferase 2 (CPT2) at K239, enhancing its ubiquitination and reducing its protein stability. This subsequently inhibits fatty acid oxidation (FAO) and reactive oxygen species (ROS) production, thereby protecting the heart against the progression of diabetic cardiomyopathy (Guo et al., 2025).

In adipose tissue, FOXO transcription factors function as important downstream effectors of the insulin/IGF signaling pathway. This study revealed that SIRT2 promotes lipolysis and inhibits adipocyte differentiation in adipose tissue in response to nutritional deprivation and energy expenditure. The mechanism by which this occurs involves SIRT2 deacetylating FOXO1, thereby promoting its binding to PPARγ and suppressing the transcriptional activity of PPARγ, thus regulating lipid metabolism and energy homeostasis (Wang and Tong, 2009).

Recent studies have shown that hypoxia-inducible factor 1α (HIF1α) suppresses SIRT2 expression in visceral white adipocytes and reduces the deacetylation of peroxisome proliferator-activated receptor gamma coactivator-1α (PGC-1α), thereby inhibiting fatty acid β-oxidation and mitochondrial gene expression, ultimately promoting obesity and associated metabolic abnormalities (Krishnan et al., 2012).

Collectively, these findings indicate that SIRT2 coordinates lipid metabolism through tissue-specific regulation of key metabolic targets, thereby contributing to cellular energy homeostasis. However, the effects of SIRT2 on lipid metabolism vary across tissues and metabolic contexts, and the basis for these substrate-specific responses remains incompletely understood. In addition, whether SIRT2-mediated lipid metabolic regulation directly contributes to aging and lifespan remains to be determined. Future studies using tissue-specific models and integrating metabolic profiling with aging-related phenotypes will help define the contribution of SIRT2-regulated lipid metabolism to metabolic health and aging.

### 3.3 Regulation of mitochondrial metabolism

Mitochondria are central to cellular metabolism, integrating nutrient oxidation to support ATP production while contributing to biosynthetic processes and redox homeostasis. Mitochondrial metabolic dysfunction has been implicated in a wide range of diseases (Spinelli and Haigis, 2018). Emerging evidence indicates that SIRT2 preserves mitochondrial metabolic function through the regulation of mitochondrial bioenergetics, redox homeostasis, mitochondrial dynamics, and mitochondrial quality control, thereby supporting cellular energy homeostasis.

Oligodendrocytes deliver the NAD-dependent deacetylase SIRT2 to the axons of neurons via exosomes, promoting the deacetylation of mitochondrial ANT1/2 to enhance ATP production, thereby maintaining and strengthening energy metabolism in the axons (Chamberlain et al., 2021). Just as SIRT2 plays a role in promoting ATP production by mitochondria, it also plays an essential role in maintaining the integrity of mitochondria in vascular endothelial cells.

SIRT2, a member of the sirtuin family (SIRT1–7), functions as a tubulin deacetylase, is involved in the cellular stress response, and is highly expressed in vascular endothelial cells. Studies have shown that inhibition or knockdown of SIRT2 in vascular endothelial cells leads to a decrease in mitochondrial membrane potential and intracellular ATP levels, accompanied by an increase in apoptosis and necrosis. These findings suggest that SIRT2 plays a crucial role in maintaining energy metabolism homeostasis and cell survival in porcine vascular endothelial cells (PIECs) (Zhang et al., 2016).

Beyond maintaining mitochondrial bioenergetics, SIRT2 also contributes to the maintenance of mitochondrial redox homeostasis by limiting excessive mitochondrial ROS production (Figure 1). SIRT2 is a deacetylase closely associated with age-related vascular ageing, and its activity declines with age. Deficiency of SIRT2 exacerbates vascular dysfunction and pathological vascular remodeling in aged mice. The mechanism by which this occurs is that SIRT2 deacetylates the age-associated protein p66Shc, thereby suppressing its activation and maintaining vascular redox homeostasis, which in turn reduces the production of mitochondrial reactive oxygen species (mROS). In contrast, SIRT2 deficiency leads to abnormal activation of the p66Shc–mROS axis, accelerating vascular ageing (Zhang et al., 2023; Chen et al., 2024). In addition to regulating mitochondrial redox homeostasis, SIRT2 also participates in mitochondrial dynamics and metabolic remodeling.

SIRT2 plays a crucial role in somatic cell reprogramming by regulating glycolysis and mitochondrial dynamics. The mechanism involves the downregulation of SIRT2 during somatic cell reprogramming, which induces non-canonical acetylation of mitogen-activated protein kinase kinase-1 (MEK1) at Lys175 and serine/threonine protein kinase (AKT1) at Lys20, thereby activating the extracellular signal-regulated kinase (ERK) and AKT1 signaling pathways. This ultimately leads to the activation of dynamin-related protein 1 (DRP1), which regulates mitochondrial dynamics and metabolic reprogramming (Cha et al., 2021).

In order to maintain mitochondrial function as we age, it is necessary to combine efficient mitochondrial remodeling with mitochondrial quality control. Recent studies have revealed that during cellular senescence, reduced SIRT2 activity impairs tubulin deacetylation, resulting in excessive tubulin acetylation. This alteration disrupts microtubule dynamics, impairs microtubule-dependent mitochondrial repair and mitophagy, and ultimately promotes the progression of cellular senescence (Cui et al., 2026).

Taken together, these findings indicate that SIRT2 regulates mitochondrial homeostasis at multiple levels, including energy metabolism, mitochondrial ROS production, and mitochondrial dynamics. Such regulation may support cellular homeostasis under physiological conditions, whereas alterations in SIRT2-dependent mitochondrial regulation may have distinct effects depending on the tissue and disease context. Further studies are needed to clarify the context-dependent effects of SIRT2 on mitochondrial function and their implications for metabolic health, aging, and disease progression.

### 3.4 Nutrient-sensing signaling pathways

The latest evidence suggests that SIRT2 regulates metabolic adaptation via various nutrient-sensing signaling pathways, thereby linking nutrient availability to cellular energy homeostasis.

Dysregulation of aromatic hydrocarbon receptor (AhR) signaling and tryptophan metabolism has been associated with aging and age-related disorders. Indole-3-acetic acid (IAA), a tryptophan-derived microbial metabolite, has been reported to improve healthspan and extend lifespan in *Drosophila*, with evidence implicating an AhR–SIRT2–TOR signaling axis that influences lipid and amino acid metabolism (Cao et al., 2025). In addition to mTOR signaling, SIRT2 also regulates cellular energy sensing through the AMPK pathway (Figure 1). Nutrient availability is closely linked to aging and longevity through evolutionarily conserved nutrient-sensing pathways. During caloric or dietary restriction, changes in nutrient and energy availability activate AMPK and suppress mTOR signaling, accompanied by alterations in NAD^+^ metabolism and sirtuin activity; these coordinated responses promote metabolic adaptation, autophagy, stress resistance, and cellular maintenance, thereby contributing to the beneficial effects of dietary restriction on healthspan and lifespan (Schmauck-Medina et al., 2026). Given the established role of SIRT2 in metabolic regulation and its reported involvement in calorie restriction-related responses, SIRT2 may contribute to the integration of nutrient availability with metabolic and longevity-related processes, although whether SIRT2 directly mediates the lifespan-extending effects of dietary restriction remains to be determined (Chen et al., 2021).

AMPK is a central cellular energy sensor that coordinates metabolic adaptation under energy stress. Recent studies have demonstrated that SIRT2 plays a significant role in the onset and progression of cardiovascular disease. For example, SIRT2 promotes the activation of AMPK via the deacetylation of the kinase LKB1; however, SIRT2 deficiency reduces AMPK activity, thereby exacerbating age-related and angiotensin II-induced cardiac hypertrophy and attenuating the cardioprotective effects of metformin (Tang et al., 2017). Furthermore, SIRT2 attenuates vascular smooth muscle cell senescence by activating the AMPK/FOXO3a signaling pathway, thereby slowing the progression of vulnerable plaques (Zhang et al., 2026).

Besides AMPK signaling, SIRT2 also mediates adaptive responses to nutrient deprivation through the integrated stress response. Studies have shown that under amino acid deprivation, SIRT2 acts as a downstream effector of the integrated stress response (ISR), with its protein level being translationally upregulated through an upstream open reading frame (uORF). Subsequently, SIRT2 suppresses global protein translation by deacetylating and stabilizing 4 E-BP1, thereby promoting cellular adaptation to nutrient stress (Zi et al., 2026).

Taken as a whole, these investigations indicate that SIRT2 maintains metabolic homeostasis by regulating nutrient-sensing signalling, thereby establishing a mechanistic link between metabolic adaptation and ageing.

## 4 Mechanisms linking SIRT2-Mediated metabolic regulation to lifespan

Evidence from animal models further supports an age-dependent role of SIRT2 in maintaining neuronal and metabolic homeostasis. In 13-month-old *Sirt2^−/−^* mice, loss of SIRT2 was associated with axonal degeneration, locomotor dysfunction, mitochondrial depletion, and redox and energy imbalance, whereas axonal degeneration and locomotor deficits were not evident in young mice. These findings suggest that SIRT2 may contribute to the maintenance of neuronal and metabolic homeostasis during aging, although its precise role may be tissue- and context-dependent (Fourcade et al., 2017).

### 4.1 Oxidative stress and redox homeostasis

Redox homeostasis is essential for maintaining cellular metabolic function and limiting oxidative damage during aging. Caloric restriction and oxidative stress induce SIRT2 expression, and SIRT2 promotes FOXO3a deacetylation, thereby enhancing FOXO DNA binding and the expression of FOXO target genes, ultimately reducing cellular ROS levels (Wang et al., 2007). Oxidative stress promotes the interaction between SIRT2 and PGAM2, leading to deacetylation and activation of PGAM2 at K100, thereby contributing to NADPH homeostasis (Xu et al., 2014). In the brain, dysregulated ROS homeostasis has been implicated in developmental arrest and lifespan regulation. Research has shown that during the process of developmental arrest, reduced SIRT2 expression inhibits the deacetylation of G6PD, thereby decreasing its activity. Consequently, NADPH levels drop, and high ROS levels are maintained, contributing to the induction of developmental arrest and extended lifespan (Geng et al., 2021). Recent studies have shown that, under conditions of oxidative stress, SIRT2 promotes the de-succinylation of glutamate-cysteine ligase catalytic subunit (GCLC), thereby enhancing its enzymatic activity and glutathione synthesis, and contributing to the maintenance of cellular redox homeostasis (Chen Z. et al., 2025). In addition to desuccinylation, SIRT2 has also been reported to exhibit additional lysine deacylase activities, including demyristoylation and delipoylation (Teng et al., 2015; Wang and Lin, 2021). Biochemical and structural studies have demonstrated robust demyristoylase activity of SIRT2 (Teng et al., 2015), while subsequent work showed that SIRT2-mediated delipoylation of DLAT at K259 can modulate endogenous pyruvate dehydrogenase (PDH) activity (Xie et al., 2019). These findings suggest that SIRT2 may regulate cellular metabolism not only through deacetylation but also through other forms of lysine deacylation, although the physiological relevance of these activities remains less extensively characterized than its deacetylase function.

In addition to regulating antioxidant metabolic pathways, SIRT2 may be involved in maintaining redox homeostasis by preserving mitochondrial integrity. In the Asian honeybee (*Apis cerana*), age-associated downregulation of SIRT2 expression is accompanied by mitochondrial damage and ROS accumulation. Conversely, activation of SIRT2 promotes FOXO deacetylation, thereby enhancing PINK1/Parkin-dependent mitophagy, thereby enhancing mitochondrial quality control, reducing oxidative stress and mitigating cellular ageing (Ma et al., 2026). Chronic oxidative stress and excessive ROS accumulation can contribute to persistent inflammation and have been implicated in the development of various diseases and reduced lifespan; however, the underlying molecular mechanisms require further investigation.

### 4.2 Inflammation and immune aging

SIRT2 regulates inflammatory signaling by modulating key immune pathways. SIRT2 regulates the expression of NF-κB-dependent genes by deacetylating the Lys310 site of the NF-κB subunit p65, thereby exerting a negative regulatory effect on immune and inflammatory responses. In the absence of SIRT2, p65 is excessively acetylated at this site, leading to enhanced expression of related genes and the promotion of excessive immune and inflammatory responses (Rothgiesser et al., 2010).

SIRT2 also regulates inflammasome activation during aging. SIRT2 suppresses the abnormal activation of the NLRP3 inflammasome via deacetylation, thereby alleviating age-related chronic inflammation and insulin resistance (He et al., 2020). As ageing progresses, decreased SIRT2 expression and increased mitochondrial stress trigger abnormal activation of the NLRP3 inflammasome in hematopoietic stem cells (HSCs), accelerating the decline in HSC function. Abnormal activation of the NLRP3 inflammasome is considered a reversible factor that promotes the ageing of HSCs. Studies have shown that SIRT2 enhances the regenerative capacity of aged HSCs by maintaining their function through the suppression of NLRP3 inflammasome activation via deacetylation (Luo et al., 2019). The cGAS–STING pathway is a conserved cytosolic DNA-sensing system that plays an important role in innate immune defense and antiviral responses, but its excessive activation can induce pathological inflammation and is therefore considered an important link between innate immunity and chronic inflammation (Decout et al., 2021). Studies have shown that SIRT2 promotes the disassembly of the cGAS–G3BP1 complex by deacetylating G3BP1 at K257, K276, and K376, thereby inhibiting cGAS DNA binding and condensate formation and negatively regulating the cGAS–STING signaling pathway (Li et al., 2023). Previous studies have shown that SIRT2 acts as a metabolic regulator in tumor-reactive T Cells by targeting key enzymes involved in glycolysis, the tricarboxylic acid cycle, fatty acid oxidation, and glutaminolysis, thereby suppressing T-cell metabolism and impairing effector functions. SIRT2 deficiency or pharmacological inhibition enhances glycolysis and oxidative phosphorylation in T Cells, improving their metabolic fitness, proliferation, and effector functions and consequently enhancing antitumor activity (Hamaidi et al., 2020).

SIRT2 not only participates in metabolic regulation but also plays an important role in modulating aging-associated inflammation. During aging, SIRT2 deficiency leads to abnormal activation of multiple immune pathways, promoting chronic inflammation and contributing to tissue functional decline. Conversely, increasing NAD^+^ levels enhances SIRT2-related regulatory activity and suppresses aging-associated inflammatory responses, suggesting that the NAD^+^–SIRT2 axis plays an important role in maintaining healthy aging and tissue homeostasis (Barthez et al., 2025).

Despite accumulating evidence, the cell- and tissue-specific roles of SIRT2 in immune aging and the interplay between its metabolic and inflammatory functions remain poorly defined. Future studies should elucidate the context-dependent regulation of the NAD^+^–SIRT2 axis and evaluate its therapeutic potential for restoring immune homeostasis and promoting healthy longevity.

### 4.3 Genome stability and cellular integrity

Genomic stability is a key factor determining the lifespan of cells, and SIRT2 contributes to the maintenance of genomic stability by regulating chromatin structure, chromosome stability and DNA repair. Increasing evidence suggests that SIRT2 may play an important role in maintaining genomic stability and facilitating DNA damage repair. During yeast aging, redistribution of Sir2 away from rDNA regions reduces local silencing activity, resulting in increased non-coding RNA transcription and rDNA instability, which contributes to cellular senescence (Yamamuro et al., 2025). *Sirt^2−/−^* mice exhibit genomic instability, chromosomal abnormalities, and increased susceptibility to tumorigenesis. Mechanistically, SIRT2 loss increases H4K16 acetylation and reduces PR-Set7-mediated deposition of H4K20me1 during mitosis, thereby compromising genome stability (Serrano et al., 2013). In mammals, SIRT2 contributes to chromosomal stability through regulation of BubR1. BubR1 is a mitotic surveillance protein whose expression levels naturally decline with ageing and is associated with age-related diseases. SIRT2 maintains chromosomal stability by regulating BubR1 expression and function during aging. The SIRT2–BubR1 regulatory axis provides insights into key mechanisms and represents a potential therapeutic target for interventions against age-related diseases, particularly cancer (Pun et al., 2024).

Under conditions of oxidative stress, SIRT2 may maintain genomic stability by participating in base excision repair, thereby mitigating DNA damage caused by free radicals. Deficiency in SIRT2 leads to a reduction in antioxidant defence functions, which in turn triggers increased inflammation and accelerated cellular ageing, resulting in a shortened lifespan (Geng et al., 2025). Cardiac ageing is a major risk factor for pathological and functional changes in the heart. This study revealed that SIRT2 expression is reduced in the hearts of aged macaques. Furthermore, it was demonstrated that in cardiomyocytes derived from human pluripotent stem cells, the loss of SIRT2 leads to the hyperacetylation of STAT3; subsequently, acetylated STAT3 activates the transcription of CDKN2B, thereby inducing age-like characteristics in cardiomyocytes. In a mouse model, restoring SIRT2 expression alleviates age-related cardiac dysfunction (Ye et al., 2023). Unlike many canonical genome-stability factors that primarily function in DNA damage response and repair, SIRT2 has been shown to contribute to genome integrity through NAD^+^-dependent deacetylation of proteins involved in chromatin regulation and chromosome stability. These findings raise the possibility that SIRT2 may link cellular metabolic status to genome maintenance, although this connection and its relevance to aging remain to be established.

### 4.4 Stem cell function and tissue homeostasis

Tissue regeneration and homeostasis depend on the maintenance of stem/progenitor cell function and the regulation of age-associated tissue remodeling. Sarcopenia is a serious degenerative condition commonly seen in the elderly, characterized by muscle atrophy, increased intramuscular fat infiltration, reduced myogenic capacity, chronic low-grade inflammation, and impaired muscle function. Studies have shown that Sirt2 expression is significantly reduced in the skeletal muscle of aged male mice. In aged male Sirt2 knockout mice, intramuscular adipose infiltration was markedly increased, accompanied by reduced expression of myogenic regulators such as Pax7 and NogoA, indicating impaired myogenic capacity. These findings highlight an essential role of SIRT2 in maintaining skeletal muscle regeneration and tissue homeostasis during aging (Lee et al., 2025).

With advancing age, the accumulation of senescent cells alters the tissue microenvironment and induces chronic inflammation, thereby promoting dysregulation of tissue repair and the progression of fibrosis. In this context, SIRT2 is involved in the progression of fibrosis in the liver, kidneys and heart by regulating relevant signaling pathways. SIRT2-mediated deacetylation underlies this key mechanism and represents a potential therapeutic target for interventions against age-related fibrosis (Huang et al., 2024).

These researches suggest that SIRT2 plays an important role in maintaining tissue regenerative capacity and homeostasis during aging. However, how SIRT2 regulates stem/progenitor cell function within the metabolic and inflammatory microenvironment of aging tissues remains incompletely understood. Future studies should further elucidate the tissue-specific roles of SIRT2 in stem cell self-renewal, differentiation, and senescence, and clarify the specific contribution of its deacetylase activity to these processes (Figure 2).

**FIGURE 2:**
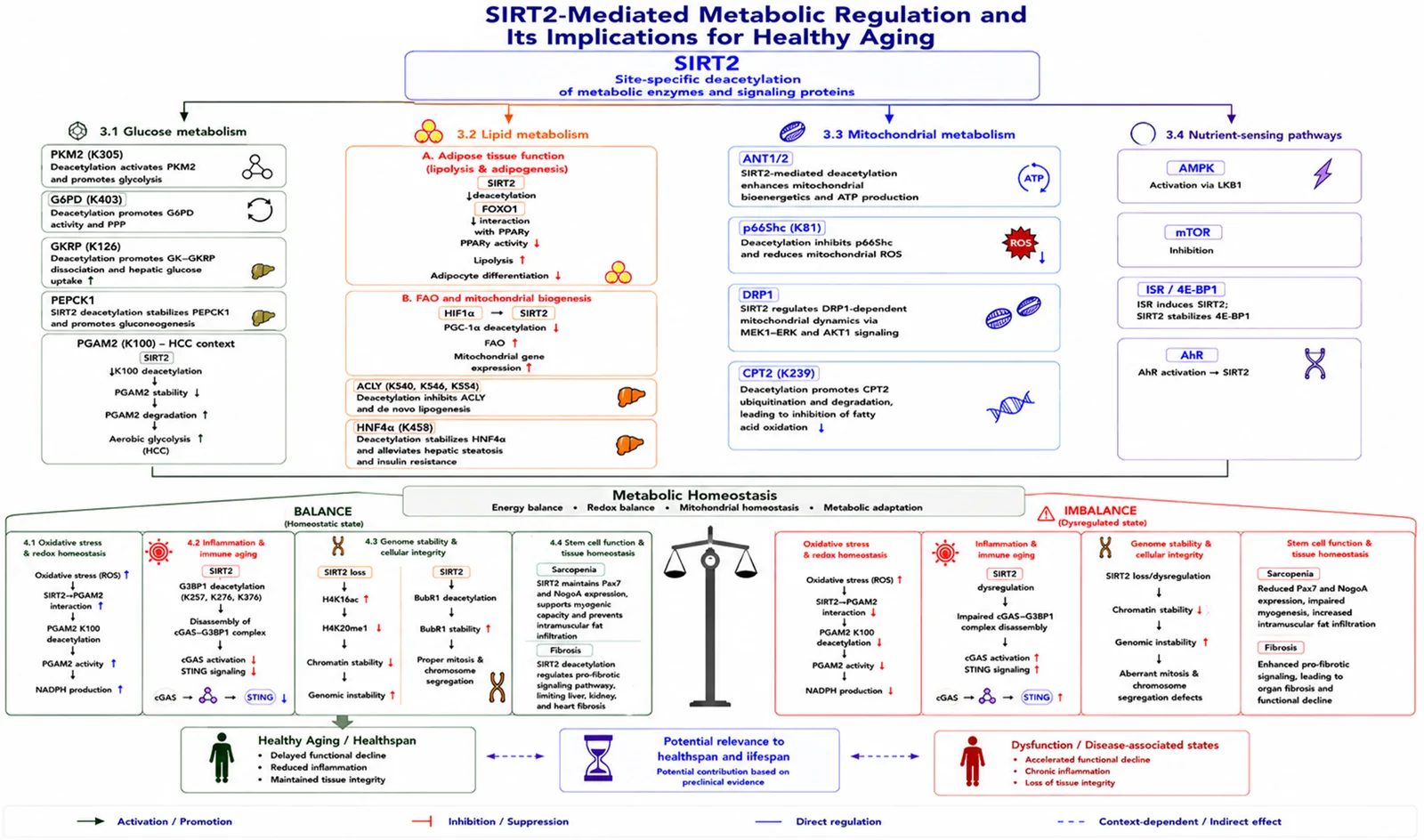
SIRT2-mediated metabolic regulation and its implications for healthy aging. SIRT2 regulates glucose, lipid, mitochondrial, and nutrient-sensing metabolism through deacetylation of multiple metabolic substrates and signaling proteins, thereby contributing to cellular metabolic homeostasis. These metabolic effects may influence redox balance, inflammatory responses, genome stability, and tissue homeostasis, with potential relevance to healthy aging and lifespan. Importantly, the functional consequences of SIRT2-mediated regulation are context-dependent and may vary across tissues, cell types, and physiological or pathological conditions.

## 5 Conclusion and perspectives

SIRT2, an NAD^+^-dependent deacetylase, functions as a metabolic regulator that links intracellular metabolic status with adaptive responses. Through site-specific deacetylation of metabolic enzymes and regulatory proteins, SIRT2 coordinates glucose, lipid, and mitochondrial metabolism, while modulating nutrient-sensing pathways and stress responses. These functions collectively contribute to the maintenance of energy homeostasis and influence aging-associated physiological changes. Beyond metabolic regulation, SIRT2 participates in multiple longevity-associated processes, including oxidative stress control, inflammatory regulation, genome maintenance, and tissue homeostasis.

By maintaining redox balance, suppressing chronic inflammation, preserving genomic integrity, and supporting tissue homeostasis, SIRT2 serves as an important molecular link between metabolic adaptation and lifespan regulation. Emerging evidence indicates that SIRT2 functions may also be regulated at the isoform level. Age-associated accumulation of SIRT2.3 in the central nervous system suggests that individual SIRT2 isoforms may contribute differently to aging-related processes, highlighting the need to further elucidate isoform-specific functions and develop precise strategies for SIRT2-targeted interventions (Sola-Sevilla et al., 2021).

Beyond its well-established deacetylase activity, the potential non-enzymatic functions of SIRT2 warrant further investigation, particularly its ability to interact with other proteins or molecules to form functional complexes and exert distinct biological effects. Given the multifactorial and complex nature of aging, a more comprehensive understanding of the regulatory mechanisms governing SIRT2 and its downstream pathways is also needed. Furthermore, determining how SIRT2 and its associated molecules can be precisely targeted or engineered for therapeutic intervention may provide new opportunities for promoting healthy aging and improving human health.
